# Human induced pluripotent stem cell-derived mesenchymal stem cells prevent adriamycin nephropathy in mice

**DOI:** 10.18632/oncotarget.21760

**Published:** 2017-10-10

**Authors:** Hao Jia Wu, Wai Han Yiu, Dickson W.L. Wong, Rui Xi Li, Loretta Y.Y. Chan, Joseph C.K. Leung, Yuelin Zhang, Qizhou Lian, Kar Neng Lai, Hung Fat Tse, Sydney C.W. Tang

**Affiliations:** ^1^ Nephrology Division, Department of Medicine, The University of Hong Kong, Queen Mary Hospital, Pok Fu Lam, Hong Kong; ^2^ Institute of Laboratory Medicine, Guangdong Medical University, Dongguan, China; ^3^ Cardiology Division, Department of Medicine, The University of Hong Kong, Queen Mary Hospital, Pok Fu Lam, Hong Kong; ^4^ Department of Ophthalmology, The University of Hong Kong, Queen Mary Hospital, Pok Fu Lam, Hong Kong

**Keywords:** induced pluripotent stem cells, mesenchymal stem cells, adriamycin nephropathy, apoptosis, fibrosis

## Abstract

Human induced pluripotent stem cell-derived mesenchymal stem cells (iPS-MSCs) are emerging as attractive options for use in cell replacement therapy, but their effect in kidney diseases remains unknown. Here, we showed that intravenous injection of iPS-MSCs protect against renal function loss in both short-term and long-term models of adriamycin nephropathy (AN). In the short-term AN model, iPS-MSCs conferred a substantial anti-apoptotic effect on tubular cells, associated with a downregulation of Bax and Bax/Bcl2 ratio and an upregulation of survivin expression. *In vitro*, conditioned medium from iPS-MSCs (iPSMSC-CM) significantly limited albumin-induced tubular apoptosis and enhanced tubular proliferation, accompanied by a reduced expression of tubular Bax and an elevated expression of Bcl2 and survivin. Oxidative stress was markedly attenuated by iPS-MSCs both in AN mice and in protein-overloaded tubular cells. In the long-term AN model, repeated injections of iPS-MSCs significantly inhibited tubulointerstitial fibrosis and reduced intrarenal deposition of collagen I, collagen IV and αSMA. Modulation of the hedgehog signaling pathway contributed to the anti-fibrotic effect of iPS-MSCs in chronic AN. Finally, we detected that most of the infused iPS-MSCs were entrapped in the lungs. In conclusion, our data support a beneficial role of iPS-MSCs in both acute and chronic AN.

## INTRODUCTION

The worldwide prevalence of chronic kidney disease (CKD) has reached epidemic proportions over the last two decades. In Hong Kong, Renal Registry data from the Central Renal Committee of Hong Kong reported that the incidence of treated end-stage kidney disease (ESRD) has risen from 95 pmp in 1996 to 150 pmp in 2013 [1]. Despite the global epidemic of CKD documented in many countries and the rising costs of treating patients with ESRD, advances in the treatment of CKD have been rather modest. Conventional management including prescription of anti-hypertensive and anti-proteinuric drugs plus stringent glycemic control may not adequately retard the progression of CKD to ESRD. Many patients progress inexorably towards ESRD and cardiovascular (CV) complications despite optimal medical therapy. On the other side, acute kidney injury (AKI) affects millions of people per year yet no therapeutic intervention has been shown to prevent the different forms of AKI or accelerate recovery from injury [2]. Novel therapeutic approaches for all forms of human kidney disease are desperately needed.

The advent of human induced pluripotent stem cell (iPS) technology brings new hope for ESRD patients who are on the long kidney transplant waiting list [3, 4]. Over the past decade, however, slow progress has been made toward the clinical implementation of iPS cells on treating kidney diseases. There are a number of unresolved issues surrounding translational research using iPS cells, among which the high risk of tumorigenicity and unforeseeable spontaneous differentiation of the transplanted cells stand out [5]. Pre-differentiation of iPS cells into adult multipotent stem cells with less proliferation capacity and restricted differentiation potential prior to transplantation might be a robust alternative approach to leverage its strengths and minimize its safety concerns. To this end, a plethora of successful cases has been reported in pre-clinical settings [6–8]. Of those iPS derivatives reported, mesenchymal stem cells derived from iPS cells (iPS-MSCs) might be the most attractive cell type for treating kidney diseases, given all current documented data invariably pointing to a beneficial role of MSCs from various sources in preventing kidney injuries [9, 10]. Unlike traditional MSCs whose accessibility are very limited due to the tedious MSC procurement procedure and the paucity of MSC population in the tissue, the source of iPS-MSCs are inexhaustible because iPS cells can theoretically be induced from any somatic cells in the body and they display unlimited proliferation capacity. Currently, no studies have yet been conducted to systemically examine the effect of iPS-MSCs on kidney diseases, despite their great success in treating other diseases, including hind-limb ischemia [11, 12], periodontitis/periodontal defect [13, 14], allergic airway inflammation [15], cigarette smoke-induced lung damage [16] cardiomyopathy [17] and bone defect [18].

To evaluate the therapeutic potential of human iPS-MSCs on AKI and CKD, we created a short-term adriamycin-induced nephropathy (AN) model featuring acute tubular injury and a long-term AN model with intrarenal fibrotic changes that resemble human CKD in immunodeficient mice. Our data support a protective role of iPS-MSCs in AN-induced tubular apoptosis and tubulointerstitial fibrosis by suppressing oxidative stress and interstitial cell proliferation. Our *in vitro* and *in vivo* imaging data further reveal that these effects might be mediated by a paracrine mechanism.

## RESULTS

### Human iPS-MSCs preserve renal function in the short-term AN model

Progressive loss of renal function is one of the major manifestations in animals with AN [19]. To determine the impact of iPS-MSCs on renal function, we transplanted iPS-MSCs into an AN model of NOD/SCID mice induced by a single injection of 5.5 mg/kg body weight ADR. At day 7, an elevation of UACR and BUN accompanied by a loss of body weight, kidney weight and serum albumin were detected (Table 1). AN mice that received human iPS-MSC transfusion had significantly lower BUN levels and higher serum albumin along with a gain of body weight and kidney weight (Table 1). Compared to AN mice given vehicle injection, only a trend toward reduction in UACR was observed in AN mice given iPS-MSC therapy (Table 1).

**Table 1:** Physical and biochemical parameters of experimental animals (at day 7 of 5.5 mg/kg ADR injection)

Parameters	Ctrl (n=5)	iPS-MSCs (n=5)	ADR (n=6)	ADR+iPS-MSCs (n=5)
Body weight (g)				
baseline	27.1±2.0	25.5±2.2	25.0±1.0	25.4±2.2
day 7	27.5±1.1	27.6±2.1	18.7±1.8^a^	21±1.6^b^
Kidney weight (mg)				
right	189.4±22.9	204.7±24.4	146.5±12.3^a^	162.5±12.4^b^
left	178.6±20.3	194.1±17.6	135.7±11.9^a^	149.7±7.5^b^
UACR (ug/mg)				
baseline	41.2±6.8	46.5±10.1	45.3±10.5	42.5±7.5
day 7	51.9±28.7	43.5±8.8	1393.0±1594.0	197±142.3
BUN (mg/dl)				
baseline	21.3±1.0	20.9±3.0	21.8±1.2	21.6±2.0
day 7	19.0±1.5	18.5±1.3	25.1±3.6^a^	17.5±1.8^b^
Serum albumin (g/l)				
baseline	70.0±4.0	67.9±4.4	63.8±7.0	69.9±5.8
day 7	68.5±6.5	67.0±4.6	26.8±6.2^a^	39.9±11.3^b^

UACR: urinary albumin-to-creatinine ratio; BUN: blood urea nitrogen.

^a^p<0.05 versus corresponding Ctrl group

^b^p<0.05 versus corresponding ADR group

### Human iPS-MSCs prevent tubular apoptosis and oxidative damage in the short-term AN model

**Figure 1:**
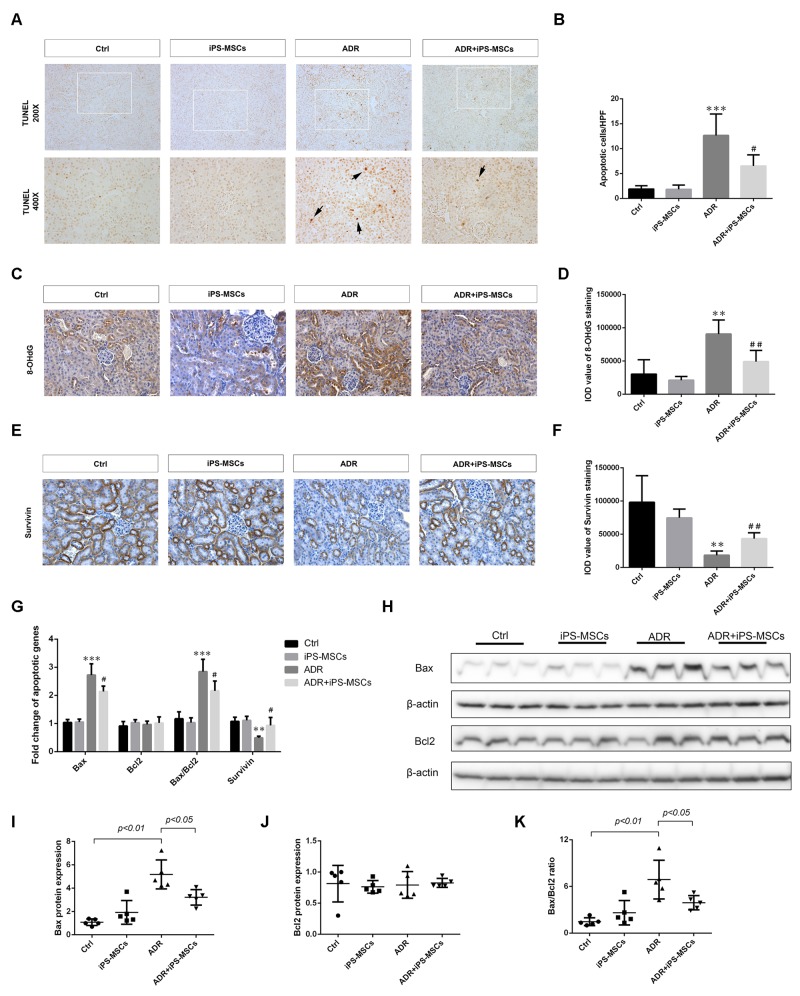
iPS-MSCs reduced tubular apoptosis and oxidative damage in murine adriamycin nephropathy (AN) **(A)** TUNEL-positive cells (arrows) revealing tubular apoptosis in mouse kidney at day 7 of ADR injection. **(B)** Quantification of apoptotic cells in renal cortex. n=5 per group. **(C)** Representative micrographs of 8-OHdG staining demonstrated that ADR-induced oxidative damage was attenuated by iPS-MSCs. Original magnification ×400. **(D)** Quantitative analysis of 8-OHdG staining. n=5 per group. **(E)** Immunohistochemical staining of survivin protein. Original magnification ×400. **(F)** Quantitative data of survivin staining. n=5 per group. **(G)** Quantitative determination of pro-apoptotic and survival gene expression in renal cortex at day 7 by real-time PCR. Protein expression was assessed by Western blot. Representative micrograph **(H)** and quantitative data **(I-K)** were shown. n=5 per group. ^**^p<0.01 and ^***^p<0.001 versus Ctrl; ^#^*p<0.05* and ^##^*p<0.01* versus ADR.

As renal tubular apoptosis is frequently reported in adriamycin-induced nephrosis [20], we next determined whether iPS-MSCs played a role in preventing renal tubular programed cell death. TUNEL assay showed that a significant amount of cells predominantly residing in renal tubules underwent apoptosis after 7 days of ADR injection, while iPS-MSCs remarkably rescued these cells from apoptosis (Figure 1A and 1B). There was less positive staining of 8-OHdG, an oxidative stress marker, in renal tubules of the iPS-MSCs treated group (Figure 1C and 1D). To elucidate the molecular basis of the anti-apoptotic effect elicited by iPS-MSCs, we detected survival and pro-apoptotic gene expressions in the renal cortex. Downregulated expression of survivin protein in AN was rescued by iPS-MSCs (Figure 1E and 1F). We next performed real-time PCR and Western blot to quantify the mRNA and protein expression of selected apoptotic genes. As shown in Figure 1G, AN triggered overexpression of Bax and downregulation of survivin mRNA in renal cortical tissues, and iPS-MSCs significantly reverted these molecular changes. The protein induction of Bax in ADR-injured kidneys was also prevented by iPS-MSCs (Figure 1H and 1I). In addition, iPS-MSCs reduced Bax/Bcl2 ratio at both transcriptional and translational levels in AN mice (Figure 1G–1K).

### Conditioned medium from human iPS-MSCs ameliorates proximal tubular apoptosis and oxidative stress induced by albumin overload *in vitro*

**Figure 2:**
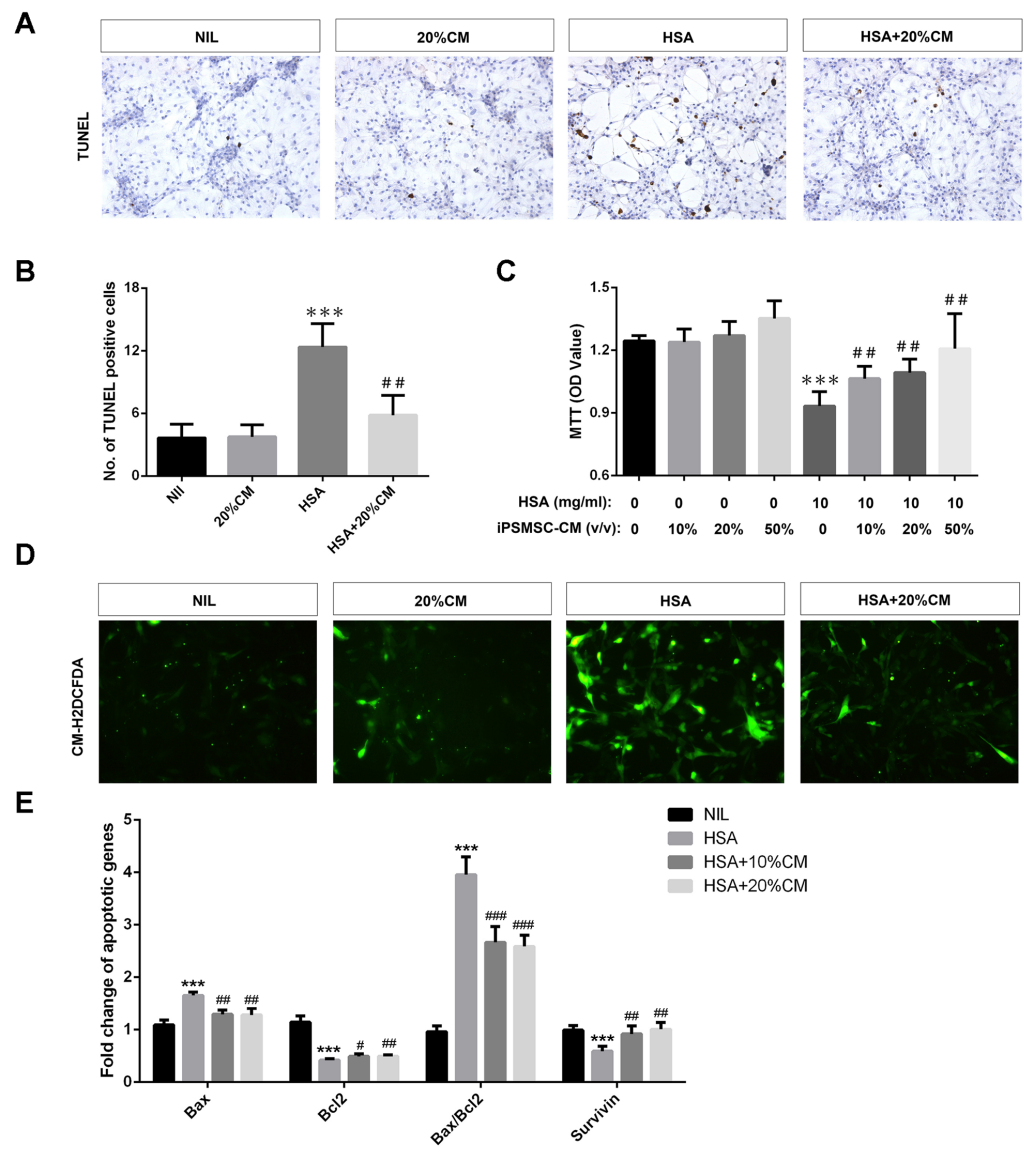
iPS-MSCs reduced apoptosis, potentiated proliferation and attenuated oxidative stress in albumin overloaded PTECs PTECs were treated with 10mg/ml human serum albumin (HSA) for 3 days in standard medium or medium supplemented with 20% iPSMSC-CM. TUNEL assay indicated that iPSMSC-CM reduced the number of apoptotic tubular cells upon albumin challenge. Representative graphs **(A)** and quantification **(B)** were presented. n=6 from three independent experiments. **(C)** MTT assay: cells were incubated with 10 mg/ml HSA for 1 day in standard medium or medium supplemented with 10%, 20% or 50% iPSMSC-CM. n=6 from three independent experiments. **(D)** Oxidative stress assay determined by absorbance of CM-H2DCFDA. Green signals indicate retention of CM-H2DCFDA in tubular cells. Original magnification ×400. **(E)** Pro-apoptotic and anti-apoptotic genes detected by real-time PCR. Data were obtained from three independent experiments. ^***^p<0.001 versus NIL or vehicle; ^#^*p<0.01,* ^##^*p<0.01* and ^###^*p<0.001* versus HSA.

Because renal tubular epithelium was the primary site where iPS-MSCs exerted their anti-apoptotic effect according to our *in vivo* data, we speculated that renal proximal tubular epithelial cells (PTECs) might be the target cells that directly benefited from iPS-MSC therapy. We tested this hypothesis by incubating human PTECs with 48-h conditioned medium collected from iPS-MSCs (iPSMSC-CM) challenged with albumin-overload. Consistent with the *in vivo* data, the degree of PTEC apoptosis induced by albumin was significantly attenuated in the presence of iPSMSC-CM by manually counting the TUNEL positive cells (Figure 2A and 2B). Of particular interest, MTT assay demonstrated that the impairment of tubular proliferation induced by albumin load was also rescued by iPSMSC-CM (Figure 2C), implying a dual role of iPSMSCs in regulating tubular apoptosis and proliferation. To measure oxidative stress in PTECs, we labeled the cells with a fluorescent probe CM-H2DCFDA. In line with the anti-oxidative action of iPS-MSCs observed *in vivo*, iPSMSC-CM significantly hampered the robust generation of ROS induced by human serum albumin (HSA) in PTECs (Figure 2D). In addition, iPSMSC-CM substantially inhibited tubular overexpression of Bax mRNA and downregulation of Bcl2 mRNA and hence reduced the upregulated Bax/Bcl2 ratio elicited by HSA (Figure 2E). Finally, iPSMSC-CM restored survivin mRNA expression under the albumin overloaded milieu (Figure 2E).

### Human iPS-MSCs limit renal function decline and morphological changes of renal cortex in the long-term AN model

**Figure 3:**
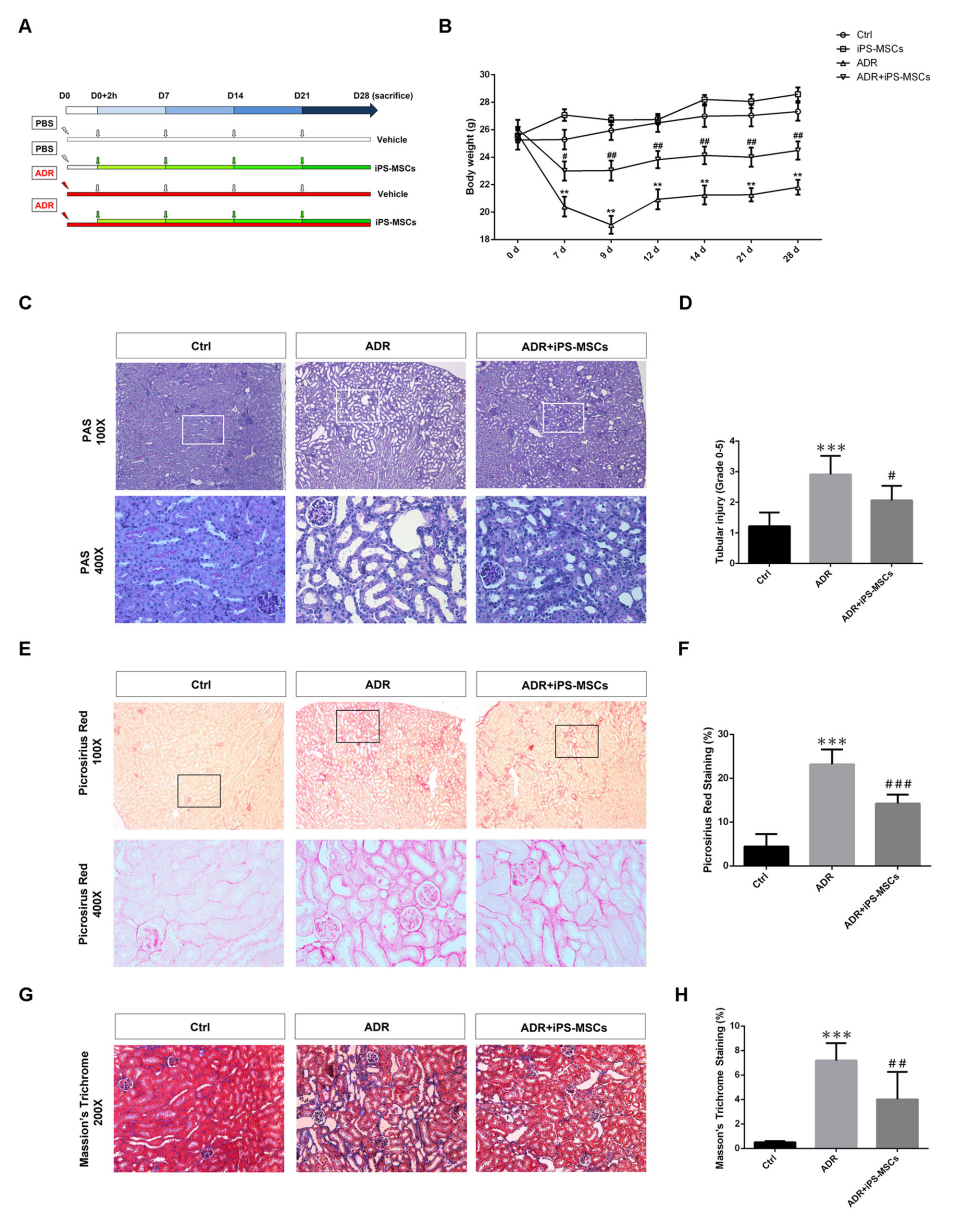
iPS-MSC treatment maintained tubulointerstitial architecture in renal cortex during AN injury **(A)** Schematic diagram of experimental design. **(B)** Body weight was measured from each indicated time point. n=6 for Ctrl, n=5 for iPS-MSCs, n=8 for ADR and n=7 for ADR+iPS-MSCs. **(C)** PAS staining showed that tubular damage in ADR mice was prevented by iPS-MSC therapy. **(D)** Quantification of tubular injury. Tubular injury was assessed by the percentage of tubular dilatation, cast formation and loss of brush border. Scoring was performed on 10 high power fields from each mouse in an observer-blinded fashion. **(E)** Picrosirius red staining indicated amelioration of tubulointerstitial fibrosis in ADR mice after iPS-MSCs infusion. **(F)** Quantification of collagen deposition in tubular interstitium (Picrosirius red staining). Data were expressed as percentage of fibrotic area (stained in red). **(G)** Representative micrograph of Masson’s Trichrome staining. **(H)** Quantification of collagen deposition in tubular interstitium (Masson’s Trichrome staining). Data were expressed as percentage of fibrotic area (stained in blue). n=5 for Ctrl, n=8 for ADR and n=7 for ADR+iPS-MSCs. ^**^p<0.01 and ^***^p<0.001 versus Ctrl; ^#^*p<0.05*, ^##^*p<0.01* and ^###^*p<0.001* versus ADR.

As chronic exposure of mice to ADR leads to progressive decline in renal function and destruction of renal tubules characterized by tubulointerstitial fibrosis [19], we sought to explore the therapeutic effect of iPS-MSCs on these aspects in the long-term AN model. Initially, we employed a dose of 5.5 mg/kg ADR to create a long-term model but most of the NOD/SCID mice were unable to survive >10 days with this dosage (data not shown). When the dose was reduced to 4.0 mg/kg, all mice remained viable for 28 days. The experimental design was depicted in Figure 3A. Similar to previous study on SCID mice [21], body weight of NOD/SCID mice given the lower dose of ADR injection dropped progressively over the first 9 days, reaching a nadir by day 9, and then steadily increased, though not reaching baseline (Figure 3B). Starting from day 7, multiple injections of iPS-MSCs at weekly intervals significantly prevented the loss of body weight in AN mice (Figure 3B). Loss of kidney weight induced by ADR was also prevented by iPS-MSCs (Table 2). At day 28, ADR-injected mice developed functional features of chronic kidney disease including proteinuria, elevated serum creatinine and BUN, and hypertension (Table 2). Although a mild effect on UACR was observed, iPS-MSCs therapy markedly brought the elevated serum creatinine, BUN and systolic blood pressure down to the baseline level in AN mice (Table 2). Morphologically, iPS-MSCs reduced renal tubular damage in the long-term AN model (Figure 3C and 3D).

**Table 2:** Physical and biochemical parameters of experimental animals (at day 28 of 4.0mg/kg ADR injection)

Parameters	Ctrl (n=7)	iPS-MSCs (n=8)	ADR (n=8)	ADR+iPS-MSCs (n=7)
Kidney weight (mg)				
Right	221.9±28.7	211.9±18.5	153.5±12.1^a^	192.0±21.0^b^
Left	195.0±26.6	199.6±18.6	146.3±9.2^a^	183.9±17.3^b^
UACR (ug/mg)				
baseline	56.3±19.0	42.4±7.7	41.9±9.0	40.4±7.0
day 28	32.5±2.3	39.3±12.0	547.4±359.7	428.9±176.0
BUN (mg/dl)				
baseline	18.3±4.3	18.3±3.0	18.2±1.4	17.8±1.7
day 28	17.7±1.3	17.1±0.9	21.2±3.3^a^	17.6±2.1^b^
SCr (mg/dl)				
baseline	0.33±0.10	0.35±0.07	0.30±0.10	0.26±0.08
day 28	0.32±0.16	0.30±0.12	0.56±0.21^a^	0.23±0.16^b^
SBP (mmHg)				
baseline	110.7±7.3	112.3±3.7	111.2±4.2	109.6±3.1
day 28	110.1±7.2	111.2±3.2	127.2±15.5^a^	112.3±13.9^b^
sTSG-6 (pg/ml)				
day 28	13.7±4.9	19.0±17.2	39.7±14.6^a^	34.6±16.5

UACR: urinary albumin-to-creatinine ratio; BUN: blood urea nitrogen; SCr: serum creatinine; SBP: systolic blood pressure; sTSG-6: serum TSG-6.

^a^p<0.05 versus corresponding Ctrl group

^b^p<0.05 versus corresponding ADR group

### Human iPS-MSCs attenuate tubulointerstitial fibrosis in the long-term AN model

**Figure 4:**
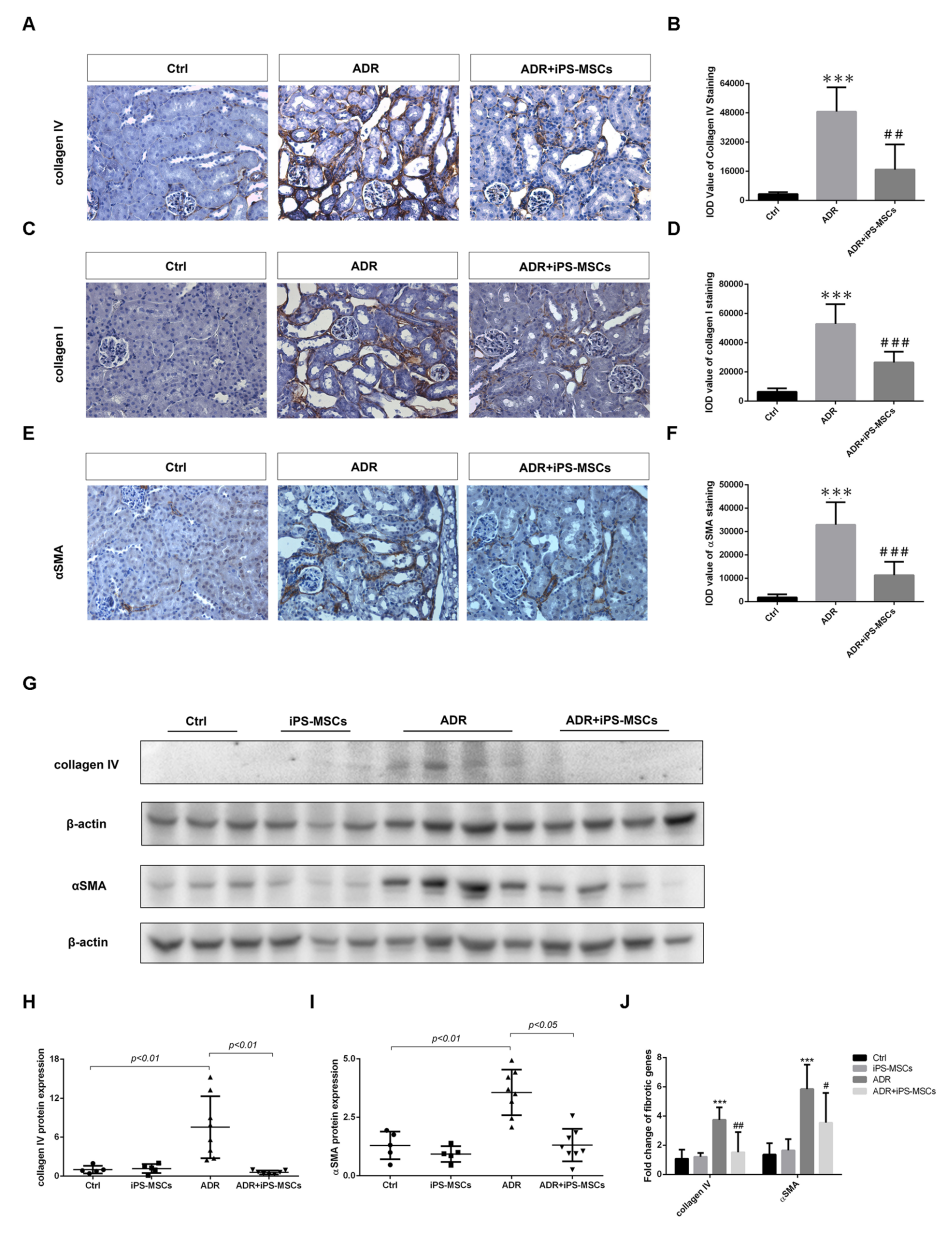
iPS-MSCs attenuated interstitial fibrosis in AN mice **(A)** Representative micrograph of immunohistochemical collagen IV staining, original magnification ×400. **(B)** Quantification of collagen IV staining. Eight field of views per slide were counted and data were expressed as IOD. Mice received PBS serve as control (Ctrl). **(C)**, **(D)** Representative photomicrographs and quantification of collagen I protein. Original magnification ×400. **(E)**, **(F)** Immunohistochemical staining and quantification of αSMA protein in tubulointerstitium. Original magnification ×400. n=5 for Ctrl, n=8 for ADR and n=7 for ADR+iPS-MSCs. ^***^p<*0.001* versus Ctrl; ^#^*p*<*0.05*, ^##^*p*<*0.01* and ^###^*p*<*0.001* versus ADR. **(G)** Western blot of pro-fibrotic proteins in renal cortex. **(H)**, **(I)** Quantitative analysis of renal cortical collagen IV and αSMA protein expression. **(J)** Real-time PCR analysis of fibrotic genes in renal cortex. Ctrl: n=5; iPS-MSCs: n=5; ADR: n=8; and ADR+iPS-MSCs: n=7. ^***^p<0.001 versus Ctrl; ^#^*p<0.05* and ^##^*p<0.01* versus ADR.

To examine the possible anti-fibrotic action of iPS-MSCs on AN, we performed picrosirius red staining to characterize collagen deposition in the tubulointerstitium. A vast area of renal fibrosis was observed in ADR injected mice. Repeated iPS-MSCs administration significantly reduced the severity of fibrosis (Figure 3E and 3F). These results were further confirmed by Masson's Trichrome staining (Figure 3G and 3H). To characterize further the deposited proteins of interstitial fibrosis, we stained sections for the fibrotic markers αSMA, collagen I and collagen IV. There is a greater positive staining of these markers in AN mice compared with control animals, which was attenuated by iPS-MSC therapy (Figure 4A–4F). Real-time PCR and Western blot analysis demonstrated that the overexpression of αSMA and collagen IV (mRNA and protein) in renal cortex of AN mice were markedly suppressed by iPS-MSCs (Figure 4G–4J).

### Inhibition of hedgehog signaling in myofibroblasts contributes to the anti-fibrotic effect of iPS-MSCs in long-term AN

**Figure 5:**
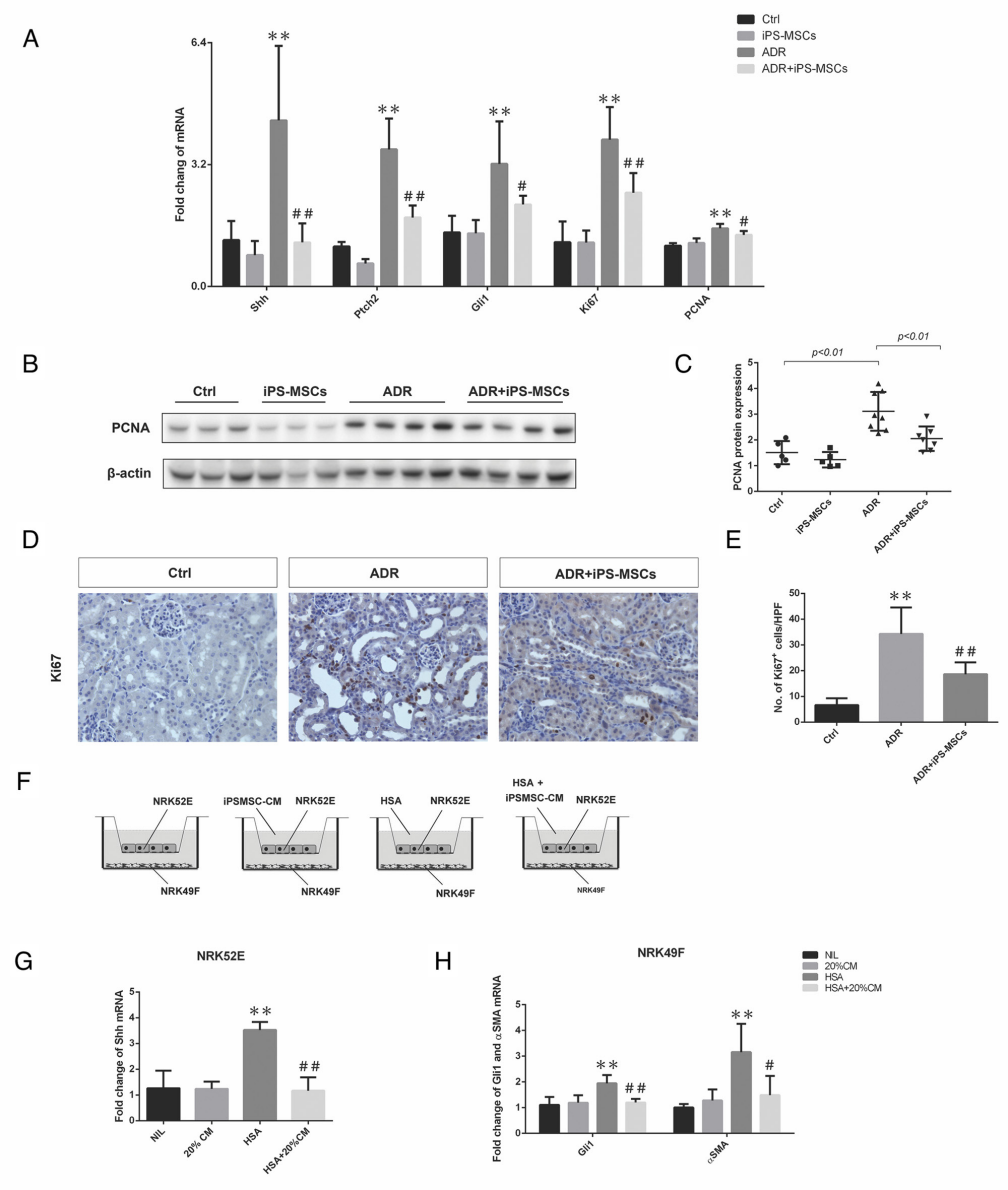
iPS-MSCs modulated fibroblast activation by attenuating hedgehog signaling pathway **(A)** Real-time PCR of Shh, Ptch2, Gli1, Ki67, and PCNA expression in the kidneys at 28 days of ADR injection (Ctrl: n=5; iPS-MSCs: n=5; ADR: n=8; and ADR+iPS-MSCs: n=7). **(B)**, **(C)** Western blot to detect PCNA expression in renal cortex and its quantification. **(D)**, **(E)** Immunohistochemical staining and quantification of Ki67 expression in tubulointerstitium. n=5 for Ctrl, n=8 for ADR and n=7 for ADR+iPS-MSCs. **(F)** Co-culture design to test the interaction of NRK52E and NRK49F and the role of iPS-MSC conditioned medium. **(G)**. Real-time PCR of Shh mRNA expression in NRK52E (n=3). **(H)**. Real-time PCR of Gli1 and αSMA mRNA in NRK49F collected from co-culture. Data were collected from three independent experiments. ^**^p<0.01 versus Ctrl; ^#^*p<0.05* and ^##^*p<0.01* versus ADR.

Interstitial myofibroblast proliferation and differentiation arising from activation of hedgehog signaling has been recently linked to kidney fibrosis in a variety of animal models [22, 23]. To investigate whether the anti-fibrotic action of iPS-MSCs was mediated by modulating this signaling pathway, we detected the expression of Shh, Ptch2 and Gli1, the key component genes from hedgehog signaling, in renal cortical lysates. ADR-induced overexpression of Shh, Ptch2 and Gli1 mRNA was significantly inhibited by iPS-MSCs (Figure 5A). This brought about reduced expression of proliferative genes including Ki67 and PCNA in AN mice that received iPS-MSC transfusion compared with the untreated AN group (Figure 5A). Moreover, we further showed that overexpression of PCNA protein from the renal cortex of AN mice was markedly attenuated by iPS-MSCs (Figure 5B and 5C). By immunohistochemical staining, Ki67^+^ cells were mainly localized to the interstitial area and iPS-MSC treatment significantly reduced the number of Ki67^+^ cells (Figure 5D and 5E). As previous study in AN model has demonstrated that Ki67^+^ cells are activated myofibroblasts residing within the renal tubulointerstitium [22], our data indicated that iPS-MSCs might inhibit myofibroblast proliferation by deactivating hedgehog signaling. To further elucidate the mechanism, we devised an *in vitro* co-culture system that physically separates two cell types but allows cell-to-cell communication through secreting factors (Figure 5F). In this system, we showed that albumin overload induced Shh mRNA (a ligand of the hedgehog signaling pathway) in a proximal tubular cell line NRK52E and Gli1 mRNA (an effector gene of hedgehog signaling pathway) overexpression in a fibroblast cell line NRK49F (Figure 5G and 5H). When iPSMSC-CM was introduced to the system, both genes were significantly downregulated (Figure 5G and 5H). In addition, iPSMSC-CM partially blocked the overexpression of fibrotic gene aSMA in NRK49F. This data supports a paracrine mechanism of iPS-MSC in modulating fibrotic response by interrupting the tubulo-interstitial crosstalk.

### iPS-MSCs are entrapped in the lungs after intravenous administration

**Figure 6:**
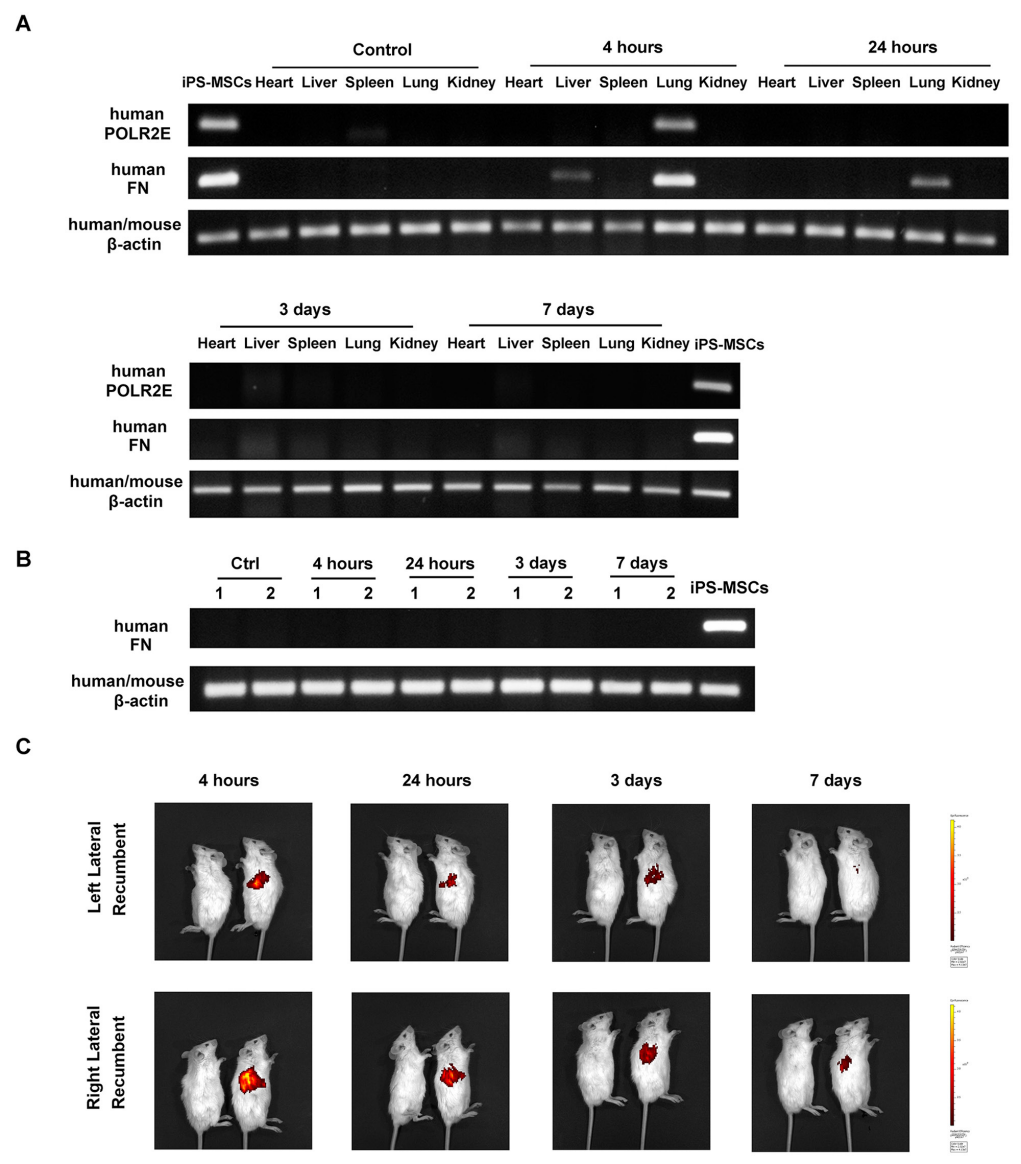
Stem cell tracking revealed trapping of iPS-MSCs in the lungs after intravenous injection **(A)** Representative agarose gels showing the expression of human POLR2E and FN mRNA in mouse heart, liver, spleen, lung, and right kidney by using specific primers. Data were representatives of 3 mice from each time point. **(B)** RT-PCR showing human FN mRNA expression in mouse left kidney at four different time points after iPS-MSC injection. Numbers (1-2) indicate individual mouse in a given group. **(C)** *In vivo* imaging of NOD/SCID mice. Mice were injected with unlabeled iPS-MSCs (left) or DiR-labeled iPS-MSCs (right).

To track the fate of stem cells after intravenous injection, we examined human gene expression in various tissues from NOD/SCID mice. Human POLR2E specific primers were used according to a previous study [24], Human POLR2E was mostly expressed in the lungs at 4 h of iPS-MSC transfusion (Figure 6A). To rule out the possibility of false negative signals from a low expression level of POLR2E gene in iPS-MSCs, we further confirmed the results with specific primers for human FN, a gene expressed more abundantly in iPS-MSCs. Again, the lung was the main tissue where FN expression was detected at 4 h of iPS-MSCs injection, and its expression persisted for 24 h albeit to a much lesser extent, and disappeared after 3 days (Figure 6A). Expression of either human POLR2E or FN was not detected in the right (Figure 6A) or left (Figure 6B) kidneys. In addition, *in vivo* imaging confirmed that the distribution of DiR-labeled iPS-MSCs was mainly confined to pleurae and no signal was detected at the site of the kidneys for up to 7 days after injection (Figure 6C). It has been shown that the protective effect of BM-MSC in myocardial infarction was mediated by TSG-6 whose release was activated when BM-MSCs were trapped in lung [25]. To examine whether the same mechanism applies to our models, we measured the serum TSG-6 level for all groups of mice at endpoint. Our data showed that serum TSG-6 molecule was not significantly elevated after repeated iPS-MSCs treatments (Table 2). Instead, we observed a remarkable elevation of TSG-6 after ADR treatment. This data indicated that TSG-6 might not be a potential candidate mediating the renoprotective effect of iPS-MSCs.

## DISCUSSION

Knowledge on the therapeutic use of MSCs in kidney diseases is growing rapidly in recent years. As a more robust cell replacement candidate, iPS-MSCs have been demonstrated to be effective in various types of diseases [11–18]. Thus far, however, little is known about the potential effect of iPS-MSCs in attenuating renal dysfunction and kidney damage due to acute or chronic injuries. We herein extended the application of iPS-MSCs to an animal model of AN. Our study is the first to report the capability of human iPS-MSCs in reversing ADR-induced acute and chronic kidney injury at multiple levels, including preserving renal function, limiting tubular apoptosis at the acute phase and reducing tubulointerstitial fibrosis at the chronic phase. We also delineated that ablation of intracellular oxidative stress and interruption of hedgehog signaling might be mechanistically related to the anti-apoptotic and anti-fibrotic effects exerted by iPS-MSCs.

Adriamycin-induced nephropathy in mice is reported to be an excellent model commonly used for pharmacological intervention studies by virtue of its capability to recapitulate the cardinal features of kidney disease including biochemical changes in serum and urine, morphologic features of glomerulosclerosis, and clinical nephrosis [26]. It is also a prevailing model to test the efficacy of stem cells on kidney disease in the past decade [27–29]. To circumvent the issue of immunorejection of exogenous human cells in mice, we chose to establish AN in NOD/SCID mice. We showed that a dose of 5.5 mg/kg ADR after 7 days ’ exposure and 4.0 mg/kg after 28 days’ exposure significantly induced albuminuria, and elevations in serum creatinine and BUN. The capability of iPS-MSCs to mitigate renal dysfunction in AN was associated with a prevention of body weight and kidney weight loss at 7 days and 28 days as well as hypertension at 28 days, strengthening a genuine renoprotective role of iPS-MSCs. However, iPS-MSCs failed to attenuate albuminuria in both models, although an insignificant trend was observed. This might be ascribed to a limited recovery of the insult to the glomerular endothelium, a primary target of ADR-mediated renal injury [30], which forms an integral component of the glomerular filtration barrier against protein leakage. Indeed, we found no significant discrepancy in the extent of glomerular damage in AN mice between iPS-MSCs treated and untreated groups (data not shown). This lack of anti-proteinuric effect was also documented by other groups using other sources of stem cells in this model [27, 28].

We have previously established the pivotal? role of tubular epithelial cells in orchestrating tubulointerstitial damage in proteinuric kidney diseases [31–33]. In AN, tubular apoptosis was shown to emerge as early as 7 days after adriamycin injection [20]. Consistent with this finding, our 7-day AN model displayed markedly more apoptotic cells mainly located in the tubular epithelium, and iPS-MSCs significantly attenuated tubular apoptosis. This anti-apoptotic effect was accompanied by decreased Bax expression, Bax/Bcl2 ratio and a restoration of survivin loss in the renal cortex of AN mice, suggesting that the intrinsic apoptotic signaling pathway might be intercepted by iPS-MSCs. *In vitro*, iPSMSC-CM remarkably limited PTEC apoptosis and promoted PTEC survival under albumin overloaded condition, with a substantial rescue of the abnormal expression of pro-apoptotic and survival genes. This anti-apoptotic action of iPS-MSCs on tubular cells might be attributed to the alleviation of oxidative stress as evidenced by our *in vivo* and *in vitro* finding that tubular ROS activation was dramatically inhibited by iPS-MSCs. As tubular apoptosis is a predominant cellular event commonly observed in various types of acute kidney disease models regardless of the primary insult [34], the tubular anti-apoptotic effect mediated by iPS-MSCs observed here might provide insights on the potential application of iPS-MSCs in other AKI models.

Another significant finding here is that tubulointerstitial fibrosis caused by ADR was also prevented by iPS-MSCs. This is reflected by the reduction of cortical deposition of total collagen at day 28 of ADR injection in mice treated with iPS-MSCs. Indeed, iPS-MSCs also interrupted the synthesis of extracellular matrix proteins such as collagen I and IV and reduced the expression of the myofibroblast marker αSMA. Previous cell-based therapies in AN have focused on the beneficial effect of stem cells on glomerular damage. For example, Manasco A. *et al* reported that MSCs reduced apoptosis of podocytes in chronic AN [27]. Similarly, Zoja C.*et al* proposed that glomerular podocytes and progenitor cells are the potential cellular targets for MSCs to exert their reparatory effect [28]. As renal function deterioration correlated better with the extent of tubulointerstitial damage than that of glomerular changes [35, 36], the anti-fibrotic effect of iPS-MSCs reported here may have provided another explanation for the recovery of renal function after stem cell injection.

It has long been considered that epithelial-to-mesenchymal transition contributes significantly to tissue fibrosis [37], only to be questioned by recent studies [38, 39]. Interestingly, a theory of tubulointerstitial cross-talk has been proposed recently [40, 41]. This theory highlighted that signaling molecules secreted from tubular epithelial cells activate interstitial myofibroblasts to proliferate and secrete extracellular matrix proteins [42]. In this context, hedgehog signaling was heavily implicated in modulating myofibroblast activation in chronic kidney diseases [43]. Here, we showed *in vivo* that iPS-MSCs significantly attenuated the expression of several critical components of hedgehog signaling, and downregulated proliferative protein expression. Immunostaining of Ki67 defines the interstitial localization of proliferating cells of probable myofibroblast origin. These findings demonstrate the anti-fibrotic role of iPS-MSCs to be mediated via attenuation of the hedgehog signaling pathway.

The molecular mechanism of how iPS-MSCs suppress oxidative stress and hedgehog signaling in AN remains unknown. A possible explanation is that iPS-MSC might secrete paracrine factors into the blood stream that act on proximal tubule to mitigate the aberrant induction of oxidative stress and hedgehog signaling pathway. This paracrine hypothesis has been supported by many other studies using iPS-MSCs [11-13, 17]. Our *in vitro* data also partially supported this mechanism as conditioned medium from iPS-MSCs exerted similar effects on reducing oxidative stress and thus tubular apoptosis. In addition, our molecular tracking technology combined with *in vivo* imaging showed that most of the iPS-MSCs after infusion were trapped in the lungs, and could not be detected in the kidney at any given time point. This observation is in accordance with a previous study showing an absence of transplanted MSCs in the kidney without undermining their effect on ischemic AKI [44]. Indeed, this lung barrier phenomenon for intravenous cell transplantation has been documented extensively in the literature [45]. Pulmonary entrapment would probably reduce the number of stem cells homing to non-pulmonary tissues such as the kidney and therefore it was initially regarded as a big obstacle to overcome in the field of cell-based therapy. Nevertheless, a later study on myocardial infarction showed that hMSCs embolized to the lungs were activated to secrete TSG-6, an anti-inflammatory factor accounting for their protective effect on the infarcted heart [25]. A more recent study also supported this theory in that pulmonary entrapment was a prerequisite for MSCs to exert their beneficial effect on peritoneal adhesion via TSG-6 [46]. As the secretome profile of iPS-MSCs is different from BM-MSCs [17], it remains unclear if TSG-6 is a key factor modulating oxidative stress and hedgehog signaling in AN. Indeed, we observed no elevated level of TSG-6 after multiple injection of iPS-MSCs. Since our *in vitro* data strongly suggest a paracrine action of iPS-MSCs on various kidney cell types (e.g. tubular cells and fibroblast), the exact renotrophic molecule(s) secreted by iPS-MSCs *in vivo* will merit further definition.

The tumorigenicity and immunogenicity of iPS cells have brought great concerns regarding their safety in clinical application [47, 48]. Some previous studies reported no signs of tumor formation when using iPS cell derivatives on other disease models [49, 50], indicating a lower tumorigenicity of the iPS cell derivatives than their parental iPS cells. Consistently, we observed no teratoma formation in this study as well as in our previous application of iPS-MSCs on another disease model [11], despite injection of cells into immunodeficient mice. However, 4 weeks’ observation is obviously not long enough to fully assess the tumorigenic potential of iPS-MSCs, and hence long-term evaluation is still required. In addition, this study was not able to assess the immunogenicity of iPS-MSCs *in vivo* due to the limitation of the existing model that applied exogenous human cells to immunodeficient mice. Although the remarkable inhibition of NK-cell proliferation and cytolytic function of iPS-MSCs [51] implied that these cells might be immune-privileged, the issue of immuno-rejection should be addressed by further studies before these cells can be clinically used in patients with kidney diseases.

In summary, we have provided novel data to support a reparatory effect of iPS-MSCs in ameliorating renal tubular apoptosis and interstitial fibrosis in murine AN. This is likely mediated by modulation of oxidative stress and hedgehog signaling through a paracrine mechanism. The anti-apoptotic and anti-fibrotic effects of iPS-MSCs warrant confirmation in other models of AKI or CKD, which could hold promise for a novel therapeutic approach in humans.

## MATERIALS AND METHODS

### Human iPS-MSCs preparation and conditioned medium collection

N1-iPS-MSC clone was used in this study. This clone was generated from iPSCs reprogramed from human fibroblasts via viral transduction with human cDNAs of Klf4, Sox2, Oct4, and c-Myc as described in our previous studies [15, 52]. MSCs were derived from iPSCs according to our established protocol [11]. In brief, iPSCs were differentiated under feeder-free condition and in DMEM medium containing 10% FCS, 10 ng/ml bFGF, 10 ng/ml PDGF, and 10 ng/ml EGF. MSCs were purified by cell sorting of CD105^+^/CD24^-^. We have verified the identity of these iPS-MSCs in our previous studies by detecting the surface marker expression and assessing their differentiation capacities [11, 15, 52]. The resulting iPS-MSCs closely resemble BM-MSCs in many regards, including the capacity of differentiation into osteoblasts, chrondrocytes and adipocytes and the expression of surface antigens such as CD44, CD49a and e, CD73, CD105, and CD166 [11, 15, 52]. iPS-MSCs within the ninth passages were used throughout this study. To collect the conditioned medium, iPS-MSCs with 70%–80% confluency were re-fed with serum-free DMEM supplemented with antibiotics. Conditioned media were harvested after 48-h incubation, centrifuged at 1000g for 5 min to remove detached cells and stored at −80°C until future use.

### Cell lines

Human primary PTECs were obtained from Lonza (Walkersville, MD, USA). The cells were cultured in Renal Epithelial Cell Growth Medium (REGM) from Lonza at 37°C in 5% CO_2_ and 95% air. Rat tubular epithelial cell line NRK52E and fibroblast cell line NRK49F were purchased from American Type Culture Collection (ATCC, Manassas, VA, USA), and maintained in DMEM/F12 medium supplemented with 5% fetal calf serum (FCS). In all experiments, there was a ‘growth arrest’ period of 24 hr in serum-free medium prior to stimulation.

### Detection of intracellular reactive oxygen species (ROS)

Detection of ROS was performed as previously described [17]. Briefly, PTECs were plated at 2×10^4^ cells/chamber on 8-well chamber slides (Nunc Lab-Tek II, Thermo Scientific) and then treated with HSA, 20% iPSMSC-CM, and HSA with 20% iPSMSC-CM. Cells in serum-free medium were served as normal control (NIL). After 24-h incubation, cells were washed with PBS and incubated with an oxidative fluorescence probe 5-(and 6-) chloromethyl-2’, 7’-dichlorodihydrofluorescein diacetate (CM-H2DCFDA, Life Technologies, Carlsbad, CA, USA) for 45 min, followed by three rounds of PBS wash and then fixed with 4% paraformaldehyde. Green fluorescent signal was visualized with a fluorescence microscope.

### Total RNA extraction and real-time PCR

Total RNA was extracted using Trizol RNA isolation reagent (Life Technologies). Reverse transcription was carried out by using High Capacity cDNA Reverse Transcription Kits (Applied Biosystems, Foster City, USA). Real-time PCR was performed on an ABI Prism 7500 sequence detection system (Applied Biosystems) using specific primers designed from known sequences in the GenBank. The primer pairs for different genes were shown in Supplementary Table 1. The mRNA level of various genes was analyzed by the SDS software (Applied Biosystems) and target values were normalized to β-actin mRNA using relative quantification method.

### Western blot analysis

Kidney tissues were homogenized and lysed with RIPA lysis buffer (Millipore, Bedford, MA, USA) containing protease inhibitor cocktails (Sigma, St Louis, MO, USA) on ice. Protein was collected after centrifugation at 12,000g and concentration was determined by Pierce BCA method (Thermo Scientific, Rockford, IL, USA). Twenty micrograms of total protein were electrophoresed through a 4-12% Bolt® Bis-Tris Plus gradient gel (Life Technologies) before transferring to a 0.2 μm PVDF membrane. The primary antibodies used in this assay were: Anti-Bax from Cell Signaling Technology (Beverly, CA, USA), anti-collagen IV antibodies from Abcam (Cambridge, UK), anti-αSMA from Sigma Aldrich (St Louis, MO, USA), anti-PCNA from Santa Cruz Biotechnology (Santa Cruz, CA, USA) and anti-β-actin from Lab Vision (Fremont, CA, USA). The membrane was incubated by secondary antibodies (Dako, Glostrup, Denmark) and detected by ChemiDoc imaging system (Bio-Rad, Hercules, CA, USA).

### Animal studies

This study was approved by the Committee on the Use of Live Animals in Teaching and Research of The University of Hong Kong and was performed in accordance with the National Institute of Health *Guide for Care and Use of Laboratory Animals*. Seven- to eight-week old NOD/SCID mice were obtained from the laboratory animal unit of the University of Hong Kong. Dose finding experiment defined an optimal dose of 5.5 mg/kg body weight of adriamycin (Pharmachemie BV, Netherlands) for short-term AN model and 4.0 mg/kg body weight of ADR for long-term AN model. For short-term acute model, AN was induced by single injection of 5.5 mg/kg ADR via tail vein. 2×10^5^ iPS-MSCs were administered intravenously at 2 hr after AN injection. Mice were sacrificed at day 7 of ADR injection. For long-term chronic model, AN was created by single injection of 4.0 mg/kg ADR via tail vein. iPS-MSCs (2×10^5^ cells/mouse) were injected intravenously into NOD/SCID mice with or without AN at 2 hr, and -repeated on day 7, day 14 and day 21 of ADR injection. Systemic blood pressure was measured by tail-cuff method. Mice were sacrificed at day 28 of ADR injection. 24-h urine samples from each group were collected by metabolic cages with access to food and water. Urinary albumin was measured by ELISA quantitation kit (Bethyl Laboratories, Montgomery, AL, USA). Blood urea nitrogen (BUN), urine and serum creatinine were determined by enzymatic method (Stanbio Laboratory, Boerne, TX, USA). Serum TSG-6 was measured by commercial ELISA kit (CUSABIO, MD, USA). Right and left kidneys from each group were collected, weighted and halved by surgical knife. One half was used for mRNA and protein collected. The other half was fixed on 10% neutral buffered formalin. Expression of pro-apoptotic and pro-fibrotic genes were assessed by real-time PCR and Western Blot.

### TUNEL assay

Apoptotic cells were evaluated by labeling and measuring DNA strand breaks in TUNEL method with an *in situ* cell death detection kit (Millipore) *as per* the manufacturer's manual. For paraffin-embedded kidney sections, apoptotic cells with positive staining were counted in 8 high-power fields (HPF) per slide. To detect tubular apoptosis under albumin challenge *in vitro*, PTECs at 2×10^4^ cells/chamber were seeded onto 8-well glass chamber slides and incubated for 3 days in medium alone, 20% iPSMSC-CM (v/v), medium supplemented with 10mg/ml HSA and 10mg/ml HSA+ 20% iPSMSC-CM. Apoptotic PTECs were counted in 8 high-power fields per group. Data were expressed with number of positive cells/HPF.

### Morphological assessment

Morphological changes of the renal cortex were determined by PAS staining on paraffin embedded sections. Tubular injury was assessed by the percentage of tubular dilation, cast formation and loss of brush border. Scoring was performed on 10 field of views (magnification: 400×) from each mice and calculated as follows: 0, none; 1, ≤10%; 2, 11%–25%; 3, 26%–45%; 4, 46%–75%; and 5, >76%. [53] Total collagen was detected by Picrosirus Red staining and Masson’s Trichrome staining kits (Sigma Aldrich).

### Immunohistochemistry

Immunohistochemistry was performed as previously described in paraffin-embedded tissue sections at a thickness of 4 μm [54]. The primary antibodies against 8-OHdG (Santa Cruz), survivin (Cell Signaling Technology), collagen IV (Abcam), collagen I (Abcam), αSMA (Santa Cruz), and Ki67 (Abcam) were used. Sections were counterstained with hematoxylin. Positive staining was quantified in 8 equivalent cortical HPFs (400×) by Image Pro Plus Software 5.0 (Media Cybernetics, Silver Spring, USA) and presented as integrated optical density (IOD).

### Co-culture set up and experimental conditions

NRK52E cells were seeded onto the 12-well transwell cassette (Corning, Cambridge, MA, USA), and grown for 24 hr until confluence. NRK49F cells (1×10^5^/well) were cultured in the lower chamber of the Transwell plate. These two cell types were physically separated by a transwell insert (of 0.4 mm pore size). HSA was supplemented into the upper chamber of transwell and incubated with or without 20% iPSMSC-CM in the upper chamber. Following 24-h co-culture, NRK52E cells from the upper chamber and NRK49F cells from lower chamber were lysed, counted, and subjected to real-time PCR to determine gene expression.

### Stem cell tracking

Total mRNA extracted from heart, liver, spleen and kidney at various time points were submitted to RT-PCR. The primer sequences specific for human POLR2E and human FN were listed in Supplementary Table 1. *In vivo* imaging was used to better visualize the tissue distribution of iPS-MSCs after transfusion. Briefly, 2×10^5^ cells were labeled with DiR (PerkinElmer, Waltham, MA, USA) before intravenous transfusion into NOD/SCID mice. Unlabeled iPS-MSCs were served as negative control. The migration and accumulation of iPS-MSCs were visually depicted and analyzed with an *in vivo* imaging system (IVIS Spectrum, PerkinElmer) at indicated time points.

### Statistical analysis

All data were expressed as means ± standard deviation unless otherwise specified. Statistical analysis was performed using GraphPad Prism v.5 for Windows (GraphPad Software Inc., San Diego, CA, USA). Differences between groups were determined using two-tailed *t* test or one-way ANOVA with Tukey's multiple comparison test.*P< 0.05* was considered statistically significant.

## SUPPLEMENTARY MATERIALS TABLE


