# Supplementary material for: Human induced pluripotent stem cell-derived mesenchymal stem cells prevent adriamycin nephropathy in mice
Source: Oncotarget. 2017 Oct 10;8(61):103640–56. doi: 10.18632/oncotarget.21760 (PMC5732756; doi:10.18632/oncotarget.21760)
Supplement: Supplementary file 1 [file oncotarget-08-103640-s001.pdf]

Human induced pluripotent stem cell-derived mesenchymal stem cells prevent adriamycin nephropathy in mice

SUPPLEMENTARY MATERIALS

Supplementary Table 1: Primers for real-time PCR and stem cell tracking

Gene	Primers
Human Bax	Forward-5'- TTCTGACGGCAACTTCAACTGG-3'<br>Reverse-5'- AGGAAGTCCAATGTCCAGCC-3'
Human Bcl2	Forward-5'-GATGGGAACACTGGTGGAGGATGG-3'<br>Reverse-5'- TCTGGAGGGCCCACGGCAG-3'
Human Survivin	Forward-5'-CTTGGCCCAGTGTTTCTTCT-3'<br>Reverse-5'-CCTCCCAAAGTGCTGGTATT-3'
Human POLR2E	Forward-5'-GCTCTGGAAAATCCGCAAGA-3'<br>Reverse- 5'-TCCTCCAGGGTCTGGTCAAG-3'
Human FN	Forward-5'-CCCAACTGGCATTGACTTTT-3'<br>Reverse-5'- CTCGAGGTCTCCCACTGAAG-3'
Human β-actin	Forward-5'-TCCATCATGAAGTGTGACGT-3'<br>Reverse-5'-GAGCAATGATCTTGATCTTCAT-3'
Mouse Bax	Forward-5'-CCCGAGCTGATCAGAACCAT-3'<br>Reverse -5'-GGGGTCCCGAAGTAGGAGAG-3'
Mouse Bcl2	Forward-5'- CTTTGAGTTCGGTGGGGTCA-3'<br>Reverse -5'- AGTTCCACAAAGGCATCCCA-3'
Mouse Survivin	Forward-5'-ATCGCCACCTTCAAGAACTG-3'<br>Reverse-5'- GGCCAAATCAGGCTCGTTCT-3'
Mouse/Rat α-SMA	Forward-5'-CTCCTCAGGACGACAATCGACA-3'<br>Reverse-5'-CCTTTCCACAGGGCTTTGTTTG-3'
Mouse collagen IV	Forward-5'-GGTCCTGTCTGGAAGAGTTT-3'<br>Reverse-5'-AAATACAATGGGAGGGAGAA-3'
Mouse/Rat Shh	Forward-5'-CAGGGGGTTTGGAAAGAGGC-3'<br>Reverse-5'- TTCTCGGCTACGTTGGGGAT-3'
Mouse Ptch2	Forward-5'- GTCCACCTAGTGCTCCCAAC-3'<br>Reverse-5'- CAGGAAGGTGCTCTGCAAGG-3'
Mouse PCNA	Forward-5'-GATGCCGTCGGGTGAATTTG-3'<br>Reverse-5'- CCATTGCCAAGCTCTCCACT-3'
Mouse Ki67	Forward-5'-CAGACTTCCACAGAGACAG-3'<br>Reverse-5'-CCTTCATCCAGATTCACAGA-3'
Mouse Gli-1	Forward-5'-CCTGGTGGCTTTCATCAACT-3'<br>Reverse-5'-ACACAGGGCTGGACTCCATA-3'
Rat Gli-1	Forward-5'-GTCACTACCTGGCCTCACAC-3'<br>Reverse-5'- CCCCTGCATTGGGGTTGTAT-3'
Mouse/Rat β-actin	Forward-5'-TCCATCATGAAGTGTGACGT-3'<br>Reverse-5'-GAGCAATGATCTTGATCTTCAT-3'
